# Efficacious genome editing in infant mice with glycogen storage disease type Ia

**DOI:** 10.1172/jci.insight.181760

**Published:** 2025-07-31

**Authors:** Benjamin Arnson, Ekaterina Ilich, Troy von Beck, Songtao Li, Elizabeth D. Brooks, Dorothy Gheorghiu, Gordon He, Matthew Weinrub, Sze Ying Chan, Hye-Ri Kang, David Courtney, Jeffrey I. Everitt, Bryan R. Cullen, Dwight D. Koeberl

**Affiliations:** 1 Division of Medical Genetics, Department of Pediatrics, and; 2 Department of Molecular Genetics & Microbiology, Duke University School of Medicine, Durham, North Carolina, USA.; 3 Department of Pediatrics, UT Southwestern Medical Center, Dallas, Texas, USA.; 4 Wellcome-Wolfson Institute for Experimental Medicine, Queen’s University Belfast, United Kingdom.; 5 Department of Pathology, Duke University School of Medicine, Durham, North Carolina, USA.

**Keywords:** Genetics, Therapeutics, Carbohydrate metabolism, Gene therapy, Genetic diseases

## Abstract

Glycogen storage disease type Ia (GSD Ia) is caused by a deficiency of glucose-6-phosphatase (G6Pase) in the liver leading to lethal hypoglycemia. Gene therapy with adeno-associated virus (AAV) vectors encoding G6Pase fails to stably treat GSD Ia early in life. We evaluated genome editing in 12-day-old infant mice with GSD Ia using 2 AAV vectors, one containing Cas9 from *Streptococcus pyogenes* and a second Donor vector that expresses a guide RNA and a *G6PC* transgene. Gene therapy with the Donor vector only was compared with genome editing using both Donor and CRISPR vectors. Treatment with genome editing (total vector dose 0.2 × 10^13^ to 2 × 10^13^ vector genomes/kg) and bezafibrate (to stimulate autophagy) was efficacious, as assessed by hypoglycemia prevention and the frequency of transgene integration, which correlated with improved survival. This therapy achieved 5.9% chromosomal transgene integration through homology-directed repair, which surpassed a threshold to prevent long-term hepatic complications. No integration was detected in the absence of the CRISPR vector. Importantly for safety, CRISPR vector genomes were depleted, and no intact, integrated CRISPR genomes were detected by long-read sequencing. Thus, genome editing warrants further development as a potentially stable treatment for human infants with GSD Ia.

## Abstract 2

Genome editing achieved a higher than expected efficacy at a relatively low dose, suggesting that it could treat rare genetic diseases early in life.

## Graphical abstract

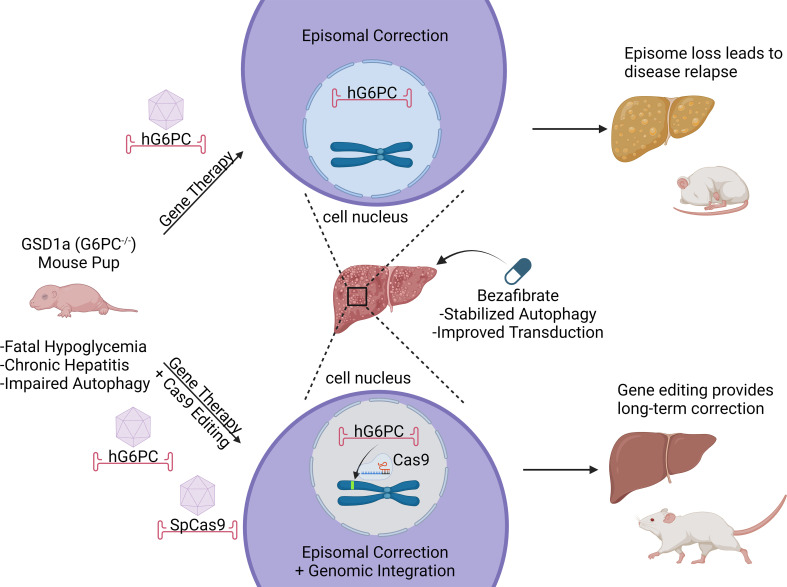

## Introduction

Glycogen storage disease type Ia (GSD Ia) is an autosomal recessive genetic disorder resulting from pathogenic variants in the glucose-6-phosphatase catalytic subunit (*G6PC*) gene (1, 2). This metabolic disease results in excess glycogen accumulation in the liver and kidneys. Patients with GSD Ia develop life-threatening hypoglycemia, as well as hepatomegaly, lactic acidosis, hyperlipidemia, and hyperuricemia. Long-term effects include impaired growth, renal failure, and hepatocellular adenomas (HCA) with a risk for hepatocellular carcinoma formation. Current therapy involves dietary supplementation with uncooked cornstarch to prevent hypoglycemia (1, 2).

Gene therapy is being explored as an alternative treatment for GSD Ia. Multiple groups have demonstrated liver-directed gene therapy that delivered a *G6PC* transgene to hepatocytes and expressed G6Pase. However, expression of the therapeutic transgene was unstable, because episomal adeno-associated virus (AAV) vector genomes were lost due to cell division in the liver of both mice and dogs with GSD Ia (3–6). Strategies to overcome this limitation of AAV vector–mediated gene therapy have included higher doses of AAV or readministration of AAV (7–9). These approaches have not been successful at preventing episomal vector genome loss and sustaining transgene expression. This limitation is particularly obvious when treating infant mice with gene therapy, as AAV vector genomes are rapidly lost from the liver early in life (7, 9–11).

Genome editing has been proposed as an alternative method to correct pathogenic variants and promote stable, long-term expression of transgenes. Initial studies in GSD Ia mice used zinc finger nucleases to insert an entire *G6PC* transgene (12), while another relied on homology-directed repair to correct a point mutation in *G6pc* (13).

This study aimed to investigate the benefit of CRISPR/Cas9-based genome editing in mice with GSD Ia. Additionally, the drug bezafibrate was administered to stimulate fatty acid oxidation and to restore autophagy in the GSD Ia liver (14), which previously increased AAV vector–mediated genome editing in the GSD Ia liver (15).

The current study evaluated the benefits of CRISPR/Cas9-based genome editing accompanied by transgene integration and expression of *G6PC* in 12-day-old *G6pc*^–/–^ infant mice with GSD Ia. Biochemical correction was evaluated by the prevention of hypoglycemia, and quantification of glycogen content. The combination of genome editing with drug treatment achieved improved survival and normalized blood glucose levels in infant mice with GSD Ia in association with transgene integration and *G6PC* expression in the liver.

## Results

### In vivo evaluation of genome editing for GSD Ia.

**Figure 1:**
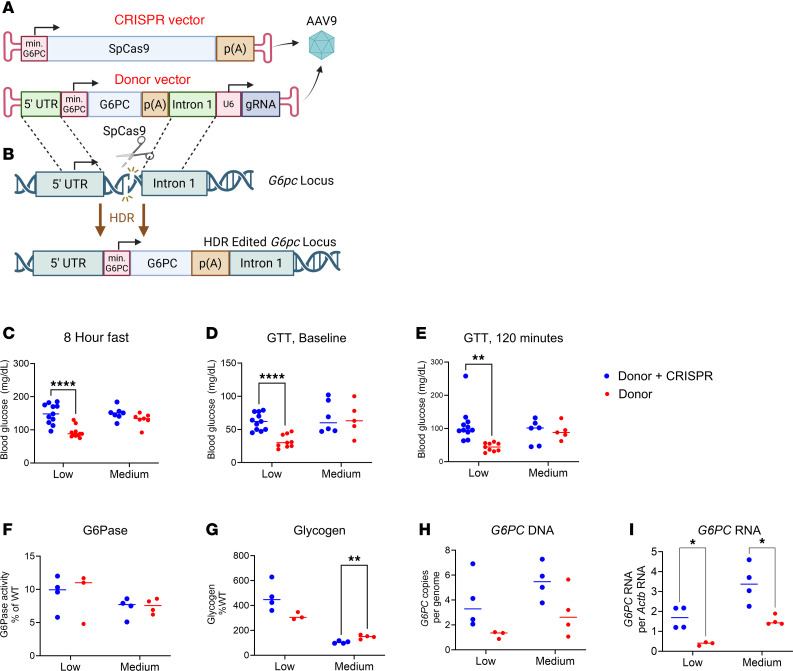
Genome editing in infant *G6pc*^–/–^ mice. (**A**) Groups treated with or without bezafibrate (Beza). Two rAAV9 vectors were administered at 12 days of age, AAV-G6PCSpCRISPR (CRISPR) and AAV-Donor (Donor), and the latter contains a human *G6PC* transgene flanked by exon 1 and intron 1 of the mouse *G6pc* gene, which permitted (**B**) integration of the transgene. Vector doses were as follows: “Low Donor + CRISPR” (*n* = 11) = Donor (2 × 10^12^ vg/kg) and CRISPR (4 × 10^11^ vg/kg); “Low Donor” (*n* = 9) = Donor (2 × 10^12^ vg/kg). “Medium Donor + CRISPR” (*n* = 6) = Donor (8 × 10^12^ vg/kg) and CRISPR (1.6 × 10^12^ vg/kg). “Medium Donor” (*n* = 5) = Donor (8 × 10^12^ vg/kg). Endpoints included (**C**) blood glucose after 8 hours of fasting; (**D**) blood glucose at baseline for GTT, and at (**E**) 120 minutes for GTT; (**F**) liver G6Pase activity and (**G**) glycogen content; and (**H**) vector DNA and (**I**) vector RNA quantification. For **F**–**H**, *n* = 4 per group, except *n* = 3 for “Low Donor.” Individual values and mean shown. *P < 0.05, **P < 0.01, ****P < 0.0001 by multiple *t* tests.

This study aimed to investigate whether genome editing from the addition of a vector containing a *Cas9* transgene (CRISPR vector) would enhance the efficacy of a vector containing the *G6PC* transgene vector (Donor vector; Figure 1). The addition of the Cas9-expressing (CRISPR) vector was expected to increase efficacy, because homology-directed repair (HDR) at the double-stranded break induced by Cas9-mediated DNA cleavage is predicted to enhance integration of the transgene, resulting in the retention of more *G6PC* transgenes and more robust G6Pase expression. Two recombinant AAV9 (rAAV9) vectors were generated for this study. The CRISPR vector contains the *Streptococcus* *pyogenes* Cas9 gene driven by a minimal *G6PC* promoter (Figure 1). The Donor vector contains a human *G6PC* transgene with minimal *G6PC* promoter flanked by homology arms consisting of the 5′ mouse *G6pc* exon 1 sequence and 3′ mouse *G6pc* intron 1 sequence. The Donor vector also contains a *U6* promoter expressing a guide RNA (gRNA) targeting the exon 1/intron 1 boundary of the endogenous mouse *G6pc* gene (Figure 1A). When CRISPR/Cas9 cleaves the murine *G6pc* locus, the transgene is predicted to insert via HDR, resulting in an integrated full-length *G6PC* transgene in the *G6pc* locus (Figure 1B). Initially, *G6pc*^–/–^ mice with GSD Ia were treated at 12 days of age with 2 different dosages of vectors: low dose (Donor 2 × 10^12^ vector genomes [vg]/kg ± CRISPR 4 × 10^11^ vg/kg), or medium dose (Donor 8 × 10^12^ vg/kg ± CRISPR 1.6 × 10^12^ vg/kg). The prevention of hypoglycemia reflects overall clinical status and the effectiveness of therapy in GSD Ia (2). Two weeks following vector administration, the *G6pc*^–/–^ mice that received the low dose of both Donor and CRISPR vectors had increased blood glucose concentrations during fasting (Figure 1C), in comparison with mice treated with low-dose Donor vector alone. A glucose tolerance test (GTT) was performed 4 weeks after treatment to further evaluate glucose metabolism. In the GTT, low-dose Donor plus CRISPR vector administration improved blood glucose at baseline following 4 hours fasting (Figure 1D) and at 120 minutes following glucose administration (Figure 1E), in comparison with Donor vector alone. Biochemical correction of the *G6pc*^–/–^ mouse livers was evaluated 4 weeks following treatment by analyzing G6Pase activity and glycogen content. G6Pase activity was similar between groups (Figure 1F). Glycogen content is a highly sensitive endpoint for biochemical correction in glycogen storage diseases (16), and the liver glycogen was significantly decreased for GSD Ia mice receiving both medium-dose Donor and CRISPR vectors, in comparison with mice receiving medium-dose Donor vector only (Figure 1G). Additionally, medium-dose Donor plus CRISPR–treated mice had hepatic glycogen content similar to the level observed for WT mice (104% ± 10% of WT). Furthermore, copies of the Donor vector trended higher in the liver for the Donor plus CRISPR–treated group, in comparison with the Donor-treated mice (Figure 1H). Expression of the Donor transgene in the liver was higher in both the low- and medium-dose Donor plus CRISPR–treated groups, in comparison with mice receiving the Donor vector only (Figure 1I), based on quantification of Donor vector RNA.

**Figure 2:**
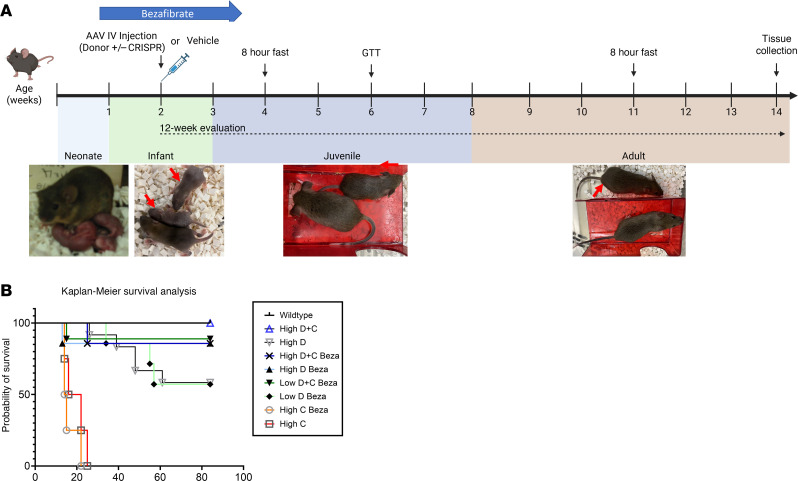
Twelve-week genome editing study. (**A**) Treatment design. The drug, bezafibrate, or vehicle was administered from day 5 to day 26 of life, and vector was administered on day 12 ± 2 of life. Tissue collection was performed at 12 weeks of age. Affected mice indicated (red arrows). (**B**) Kaplan-Meier survival analysis. Vector doses were as follows: “Low D+C” = Donor (2 × 10^12^ vg/kg) and CRISPR (4 × 10^11^ vg/kg), “High D+C” = Donor (1.6 × 10^13^ vg/kg) and CRISPR (3.2 × 10^12^ vg/kg), “Low D” = Donor (2 × 10^12^ vg/kg), “High D” = Donor (1.6 × 10^13^ vg/kg), and “High C” = CRISPR (3.2 × 10^12^ vg/kg). Groups for survival as follows: WT (*n* = 12), High D+C (*n* = 10), High D (*n* = 16), High D+C Beza (*n* = 6), High D Beza (*n* = 6), Low D+C Beza (*n* = 9), Low D Beza (*n* = 7), High C Beza (*n* = 4), and High C (*n* = 4).

Given the demonstrated benefits from the addition of the Cas9-containing CRISPR vector, the study was extended to 12 weeks to determine the most efficacious treatment. We compared high- and low-dose vector treatment, with and without administration of the drug bezafibrate. Bezafibrate is a pan-agonist of peroxisome proliferator-activated receptors (PPARs) that enhances the expression of genes involved in lipid homeostasis and energy metabolism (Figure 2A) (14). Bezafibrate has previously been shown to lower liver triglycerides and glycogen in GSD Ia mice while also increasing the transduction of AAVs and expression of transgenes (14, 15). Survival analysis revealed increased survival for GSD Ia mice receiving the Donor and CRISPR vectors at low or high dose with bezafibrate, in comparison with mice treated with high-dose Donor and no bezafibrate, or with low-dose Donor and bezafibrate (Figure 2B). Survival for both the high-dose Donor plus bezafibrate (85%) and the high-dose Donor plus CRISPR plus bezafibrate (89%) groups was incomplete, because one mouse died in each group in the first weeks following treatment, whereas the high-dose Donor plus CRISPR (100%) group had complete survival. In contrast, high-dose Donor without bezafibrate had lower survival (7 of 12; 58% survival), as did the low-dose Donor plus bezafibrate group (4 of 7; 58% survival). These data suggested that either CRISPR or bezafibrate treatment was needed to support high rates of survival. Monitoring body weight and blood testing (serum alanine aminotransferase [ALT], creatinine, cholesterol, and triglycerides) revealed a lack of toxicity from vector administration. Although GSD Ia–related differences were observed in comparison with WT mice, these abnormalities normalized by 12 weeks following vector administration (Supplemental Figures 1 and 2; supplemental material available online with this article; https://doi.org/10.1172/jci.insight.181760DS1). Liver weight was increased for all treatment groups, in comparison with WT mice, which indicated incomplete correction of the liver (Supplemental Figure 1). Additionally, mice treated with CRISPR vector only did not survive past weaning at 3 weeks of age (Figure 2B), confirming the need for a therapeutic *G6PC* transgene expression to sustain *G6pc*^–/–^ mice early in life.

### Assessment of biochemical and molecular endpoints.

**Figure 3:**
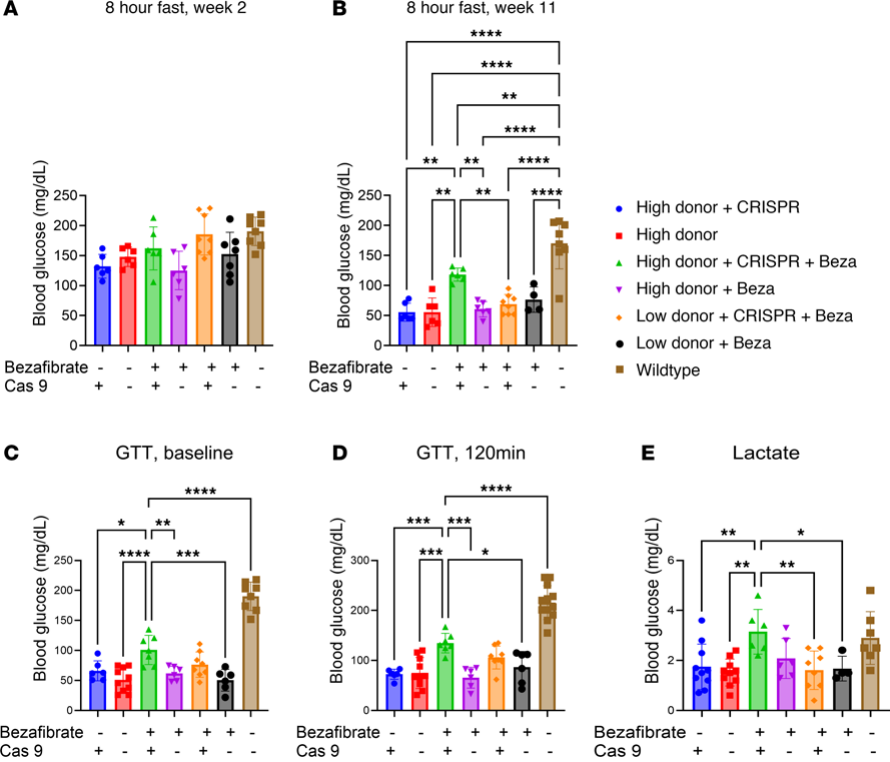
Correction of hypoglycemia. Blood glucose after 8 hours fasting performed (**A**) 2 weeks and (**B**) 11 weeks following vector administration. Blood glucose determined at (**C**) baseline after a 4-hour fast, and (**D**) at 120 minutes after dextrose administration. (**E**) Blood lactate determined 12 weeks following vector administration without fasting. Groups were as follows: WT (*n* = 8), High Donor + CRISPR (*n* = 6), High Donor (*n* = 6), High Donor + CRISPR + Beza (*n* = 7), High Donor + Beza (*n* = 7), Low Donor +CRISPR + Beza (*n* = 8), and Low Donor + Beza (*n* = 6). Individual values and mean are shown. *P < 0.05; **P < 0.01; ***P < 0.001; ****P < 0.0001 compared with High Donor + CRISPR + Beza group as determined by 1-way ANOVA with Dunnett’s multiple-comparison test.

Blood glucose concentrations during fasting were measured 2 weeks following vector administration. No differences were observed among treatment groups, and blood glucose was similar to the concentrations observed for WT mice (Figure 3A). However, by 11 weeks after treatment, the fasting blood glucose of mice receiving bezafibrate and high-dose Donor plus CRISPR vectors was significantly higher compared with all other groups of *G6pc*^–/–^ mice (Figure 3B). Blood glucose following fasting and survival did not differ based on the sex of affected mice in the different treatment groups (Supplemental Figure 3). Additionally, the GTT performed at 4 weeks following treatment revealed that the mice in the high-dose Donor plus CRISPR vectors plus bezafibrate group had elevated blood glucose at baseline and 120 minutes following glucose administration compared with all other treatment groups except bezafibrate plus low-dose Donor plus CRISPR vectors (Figure 3, C and D). Blood lactate without fasting was equivalent in week 12 of the study for the high-dose Donor plus CRISPR plus bezafibrate and WT groups, while other treatment groups had significantly lower blood lactate (Figure 3E). This trend toward lower lactate for some groups in comparison with WT mice was unexpected, because lactate is usually higher than the normal range in patients with GSD Ia (1, 2). However, this result indicated normalization for the Donor plus CRISPR plus bezafibrate group.

**Figure 4:**
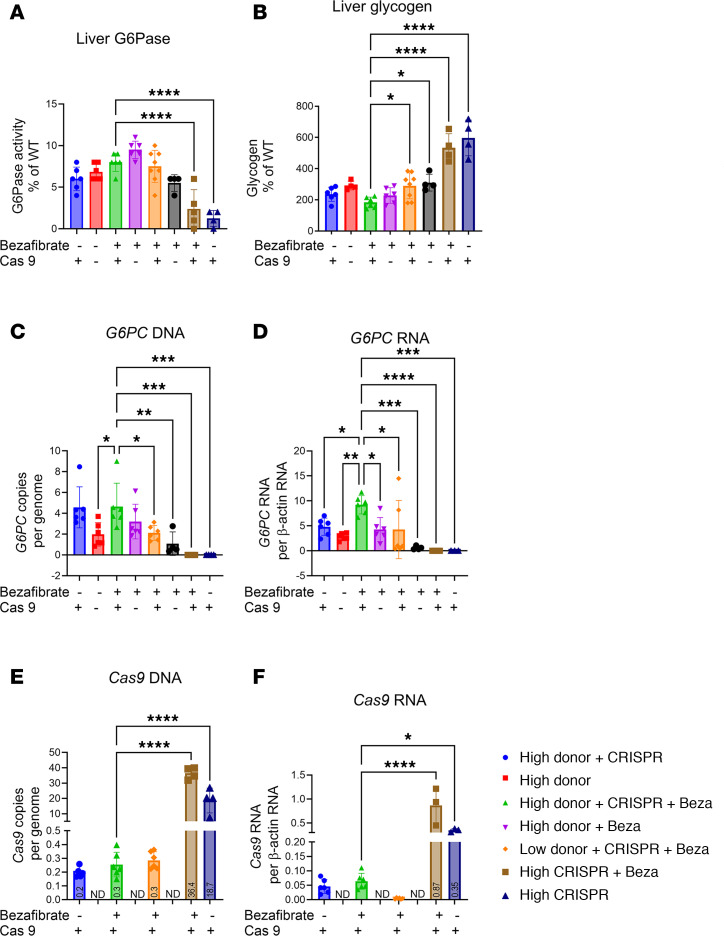
Endpoints for genome editing in the murine GSD Ia liver. Donor-treated groups collected on week 12, CRISPR-only on day 3. (**A**) Liver G6Pase. (**B**) Liver glycogen content. (**C**) *G6PC* transgene DNA. (**D**) *G6PC* transgene RNA. (**E**) *Cas9* DNA. (**F**) *Cas9* RNA. Groups were as follows: WT (*n* = 8), High Donor + CRISPR (*n* = 6), High Donor (*n* = 6), High Donor + CRISPR + Beza (*n* = 7), High Donor + Beza (*n* = 7), Low Donor + CRISPR + Beza (*n* = 8), Low Donor + Beza (*n* = 4), High CRISPR + Beza (*n* = 4), and High CRISPR (*n* = 4). Individual values and mean are shown. *P < 0.05; **P < 0.01; ***P < 0.001; ****P < 0.0001 compared with High Donor + CRISPR + Beza group as determined by 1-way ANOVA with Dunnett’s multiple-comparison test.

Analyzing the biochemical correction in the liver revealed that the administration of high-dose Donor plus CRISPR plus bezafibrate increased G6Pase activity for *G6pc*^–/–^ mice to 8.0% ± 1.1% of WT (Figure 4A). However, the G6Pase activity assay did not reveal differences between Donor vector–treated groups due to limited sensitivity. The high-dose Donor plus CRISPR vectors with bezafibrate group had the lowest liver glycogen content, which was significantly lower than for the group receiving low-dose Donor plus CRISPR vectors with or without bezafibrate (Figure 4B). Liver histology revealed decreased numbers of vacuoles associated with stored glycogen in mice treated with high-dose Donor plus CRISPR vectors, in comparison with the other groups (Supplemental Figure 4). Quantification of the Donor vector genomes revealed statistically significantly increased copy number for the high-dose Donor plus CRISPR vectors plus bezafibrate group, in comparison with groups treated with low-dose Donor or with high-dose Donor and no bezafibrate (Figure 4C). The Donor transgene RNA expression was also highest in the high-dose Donor plus CRISPR plus bezafibrate group, and significantly increased compared with all other treatment groups, confirming that this was the best treatment (Figure 4D).

Quantification of the *Cas9* transgene demonstrated low copy number at 12 weeks following treatment (Figure 4E). The administration of CRISPR vector alone did not prolong survival (Figure 2B), and tissues were collected from those groups 3 days following vector administration. Importantly, the *Cas9* transgene was 120-fold higher at 3 days following vector administration (high-dose CRISPR + bezafibrate group; 36 copies/genome), in comparison with 12 weeks (high-dose Donor + CRISPR + bezafibrate group; 0.3 copies/genome) for groups that received high-dose CRISPR vector (Figure 4E). Similarly, *Cas9* RNA was 17-fold higher at 3 days, in comparison with 12 weeks (Figure 4F). At the end of week 12, the *G6PC* transgene was sustained at 18-fold higher copy number than the *Cas9* transgene for the best-treated, high-dose Donor plus CRISPR plus bezafibrate group (Figure 4, C and E; 4.7 ± 2.2 vs. 0.25 ± 0.09), despite the Donor vector being dosed only 5-fold higher than the CRISPR vector. Furthermore, the *G6PC* RNA was 144-fold higher than the *Cas9* RNA (Figure 4, D and F; high-dose Donor + CRISPR + bezafibrate, 9.2 ± 1.8 vs. 0.07 ± 0.03). Together, these data confirmed stability of the *G6PC* transgene relative to the *Cas9* transgene.

### Assessment of immune responses.

Given the risk for immune responses to Cas9, we evaluated antibody and T cell responses without detecting evidence for anti-Cas9 responses (Supplemental Figure 5). Thus, the loss of the *Cas9* transgene could be attributed to cell division and the loss of the episomal CRISPR vector genomes.

### Evaluation of nuclease activity and transgene integration.

**Figure 5:**
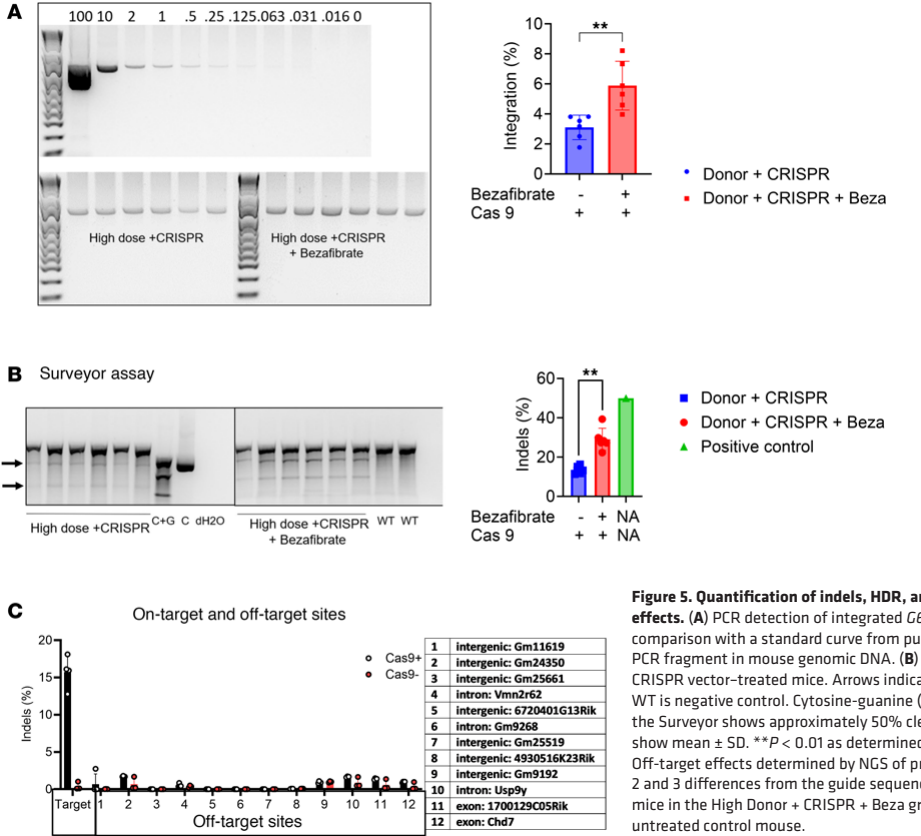
Quantification of indels, HDR, and off-target effects. (**A**) PCR detection of integrated *G6PC* transgene, in comparison with a standard curve from purified integration PCR fragment in mouse genomic DNA. (**B**) Surveyor assay for CRISPR vector–treated mice. Arrows indicate bands for indels. WT is negative control. Cytosine-guanine (C+G) control for the Surveyor shows approximately 50% cleavage. Histograms show mean ± SD. **P < 0.01 as determined by a *t* test. (**C**) Off-target effects determined by NGS of predicted sites with 2 and 3 differences from the guide sequence from 5 treated mice in the High Donor + CRISPR + Beza group and from an untreated control mouse.

Treatment with bezafibrate increased nuclease activity, as reflected by transgene integration and by the generation of indels. Bezafibrate administration increased transgene integration from 3.1% ± 0.8% to 5.9% ± 1.7% for mice treated with high-dose Donor plus CRISPR vectors (Figure 5A). Furthermore, the integrated transgene could be detected only in mice treated with Donor plus CRISPR, and no integrated transgene was observed in mice receiving the Donor vector only (Figure 5A). The Surveyor nuclease assay was used to detect indels introduced at the exon 1/intron 1 boundary of *G6pc* by CRISPR/Cas9 and repaired by nonhomologous end-joining (NHEJ). The Surveyor assay detected increased indels in the high-dose Donor plus CRISPR plus bezafibrate–treated mice (30% ± 5.6%) compared with high-dose Donor plus CRISPR alone (16% ± 2.4%; Figure 5B). No indels were detected in mice not treated with the CRISPR vector (Figure 5B, WT). These data confirmed the activity of CRISPR/Cas9 in the *G6pc*^–/–^ mouse liver, resulting in HDR-mediated transgene integration, which was increased by treatment with bezafibrate. Finally, off-target analysis was performed with next-generation sequencing of sites that were predicted to be at risk for cleavage based on homology to the guide sequence’s target site, and the presence of indels at off-target sites were similar between mice treated with CRISPR and control mice (Figure 5C).

### Long-read PacBio sequencing of transgene integration events.

**Figure 6:**
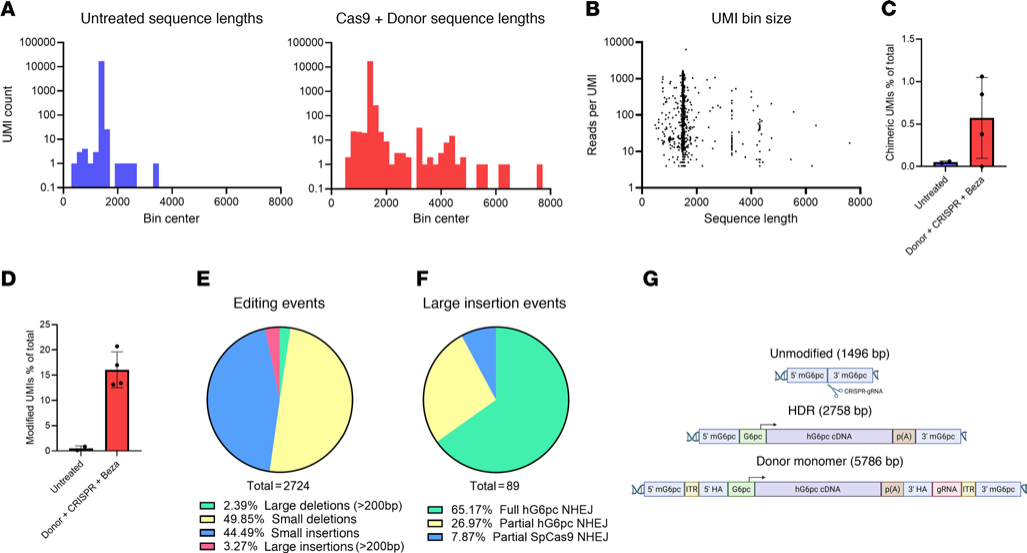
Characterization of editing outcomes by long-read PacBio sequencing. (**A**) Sequence length histograms of UMIs in liver samples from untreated (left, *n* = 4) and treated (right, *n* = 4) animals. (**B**) Scatter plot of UMIs comparing UMI read coverage to sequence length. (**C**) Frequency of chimeric UMIs detected in each sample. (**D**) Frequency of reads bearing an insertion or deletion overlapping the predicted gRNA target site. (**E**) Composition of all editing events in treated samples. (**F**) Classification of all large insertion events. (**G**) Representative schematics of unmodified, HDR-edited, and NHEJ-AAV–inserted *G6pc* amplicons.

To better understand transgene integration in liver genomic DNA, we developed long-read PacBio sequencing. This sequencing yielded 8,855,221 circular consensus sequencing (CCS) reads, which were condensed to 34,916 unique molecular identifier (UMI) pairs with a median coverage of 49 reads per UMI using the long-read umi pipeline (PMID: 33432244). While most UMIs correlated to the expected 1496 bp size of unmodified mouse *G6pc*, Cas9 plus Donor–treated animals showed an increase in both shorter and longer sequences (Figure 6A). While PCR bias can favor amplification of shorter products, there was no correlation found between the length of the sequenced product and the associated read coverage (Figure 6B). Rates of PCR chimerism were low in all samples, with marginal, nonsignificant increases in Donor plus CRISPR–treated samples (Figure 6C). Quantification of editing events identified a background rate of 0.48% in untreated samples, which is attributed to both PCR and sequencing error (Figure 6D). In treated animals, this rose to an average of 16.06% of modified alleles, ranging from 13.14% to 20.69%. Of the modified reads, most were attributable to small indels (<200 bp), with only a minority containing large insertions (Figure 6E). Of the large insertions, 58 corresponded to functional NHEJ insertions containing an intact human *G6PC* transgene, 24 contained a partial human *G6PC* transgene, and 7 contained a partial *SpCas9* transgene (Figure 6, F and G). Unexpectedly, while complete HDR events could be detected in the raw sequencing data, they lacked adequate sequencing coverage (≥3×) for UMI consensus generation (data not shown). NHEJ events included insertion of both monomeric donor episomes as well as partial concatemers containing donor-donor or donor-SpCas9 fusions.

## Discussion

This study demonstrates the efficacy of CRISPR/Cas9-based genome editing that can integrate a full-length therapeutic transgene into a defined chromosomal location through HDR in the liver of mice with GSD Ia. The highest total vector dose of 2 × 10^13^ vg/kg was relatively low, in comparison with other studies of genome editing in the liver, even those that treated neonatal mice that are most responsive to genome editing (17–21). Administering a vector that expresses Cas9, along with a Donor vector containing a functional *G6PC* transgene and gRNA, improved the therapeutic efficacy in young mice. This study demonstrated the benefit of targeted nuclease-dependent genome editing, because administering CRISPR/Cas9 improved the efficacy of the Donor transgene but had no benefits by itself. Cas9 was required to achieve appreciable transgene integration in the *G6pc* locus. This observation supports the hypothesis that HDR-mediated integration depends on nuclease activity in this model, despite reports that Donor templates can integrate spontaneously or independently of nuclease activity (18, 22). Addition of CRISPR/Cas9 increased HDR by 26-fold in mice with Crigler-Najjar (23), consistent with our data. Furthermore, adding bezafibrate, a drug known to increase transgene expression and editing efficacy, improved integration frequency and biochemical correction in mice long term (14, 15). Bezafibrate apparently increased nuclease activity, as reflected by a doubling the rate of both indel formation and HDR, although the rate of indel formation clearly exceeded the rate of HDR (Figure 5, B and C). Stable transgene integration was demonstrated in a study with the GSD Ia canine model; however, the associated biochemical improvement was minimal and attributable to the remaining episomal vector genomes (6). Similarly to previous studies of gene therapy (6), puppies with GSD Ia that were treated with genome editing as neonates eventually developed hypoglycemia and required rescue doses of gene replacement therapy (24). In the current study with *G6pc*^–/–^ mice, the combination of high-dose genome-editing vectors with bezafibrate resulted in the most efficacious outcome, as determined by the primary endpoint for clinical trials in GSD Ia (25), the prevention of hypoglycemia, which correlated with transgene integration. This observation will inform the design of future preclinical studies with the mouse or canine models of GSD Ia.

This study achieved higher rates of editing in vivo compared with previous studies in GSD Ia. The level of hepatic correction needed to successfully treat GSD I has been estimated at 3% of normal G6Pase activity (5, 26). Given the presence of approximately 4 copies of the *G6PC* transgene per mouse genome (Figure 4C) and our estimation that only approximately 6% of genomes integrated the *G6PC* transgene (Figure 5A), it is very likely that the great majority of the Donor vector genomes remain episomal and will be lost or diminished over time due to cell division in the liver (3–6). However, the demonstrated efficiency of hepatic genome editing has often been less than 1% of alleles or cells in the absence of an enhancing drug treatment or a selective advantage to increase homologous recombination (13, 18, 20, 22). Arnaoutova and colleagues developed a mouse GSD Ia model with a pathogenic missense variant in *G6pc* that was treated with genome editing using CRISPR/Cas9 to achieve HDR-mediated correction of 0.7% of alleles in the liver, which required a vector dose of 1.1 × 10^14^ vg/kg (13). In the above-mentioned canine GSD Ia study, 0.5%–1.0% of alleles were edited to contain the therapeutic transgene. The current study achieved 3.1% transgene integration for mice receiving both Donor and CRISPR vectors, which increased to 5.9% for mice that also received bezafibrate treatment (Figure 5A). The nearly 6-fold higher frequency of HDR in the current study was associated with significantly improved fasting blood glucose, increased liver G6Pase enzyme activity, and decreased liver glycogen content in *G6pc*^–/–^ mice. Furthermore, the efficacious vector dose in the current study was 5-fold lower than that administered by Arnaoutova and colleagues (13). Expression of the *G6PC* transgene expression was well above the threshold of 3% of normal G6Pase activity that prevented tumor formation from the effects of GSD Ia in *G6pc*^–/–^ mice (5, 26), given that 8% of normal G6Pase activity was demonstrated for the best-treated group (Figure 4A).

Benefits of the current genome editing strategy include the ability to treat GSD Ia without regard to the underlying pathogenic variant, as opposed to methods that will only correct a specific mutation. Importantly, the Donor vector will express the *G6PC* transgene as an episome. This feature addresses the need for high *G6PC* expression in young *G6pc*^–/–^ mice (27) to prevent mortality by including a *G6PC* promoter upstream of the transgene, which has the immediate benefit of gene therapy coupled with the long-term sustained expression of the integrated transgene.

Transgene expression from the episomal Donor vector genomes complicated the detection of benefits from genome editing, especially in the initial 4-week study. Improvement from the addition of Cas9 in the 8-hour fasting and GTT was observed in the low-dose group, but not in the medium-dose group. We speculate that no additional benefits from adding Cas9 were observed in the medium-dose groups due to higher numbers of Donor vector genomes accompanied by episomal expression, which masked any benefit from transgene integration associated with Cas9 expression. Only the medium-dose group had decreased glycogen with Cas9 at 4 weeks, but this could be attributed to low transgene expression from the low dose of Donor vector.

Extending the study to 12 weeks and adding bezafibrate confirmed the advantages of high vector dosages, and demonstrated a dose response. High-dose Donor plus CRISPR vector–treated mice had improved blood glucose during fasting, increased G6Pase activity, and decreased liver glycogen, in comparison with low-dose vector. As expected, high-dose vector administration led to more transgene copies being present in the liver accompanied by higher *G6PC* expression.

The dramatic loss of the *Cas9* transgene provides increased safety by decreasing the potential risks of prolonged nuclease activity (Figure 4, E and F). This transgene loss might be attributable to the high rate of apoptosis in the untreated GSD Ia liver (28), which also helps to explain the transience of episomal AAV vectors in GSD Ia (3–6). The Donor vector genomes could be stabilized by integration of the *G6PC* transgene, as well as a previously described potential selective advantage for corrected cells following GSD Ia genome editing (12). At the end of the study, *G6PC* transcripts were 144-fold higher than Cas9 transcripts, confirming a high therapy-to-risk ratio with regard to transgene expression (Figure 4, D and F). The risk of prolonged CRISPR/Cas9 activity has been emphasized by previous studies of integration of AAV vector genomes into double-stranded DNA breaks, further increasing the risk of persistent expression of nuclease activity from an integrated vector containing CRISPR/Cas9 (29). The current data suggest that this risk is much lower for genome editing in GSD Ia.

We performed long-read PacBio sequencing of liver genomic DNA to better characterize integration events involving vector genomes, which would detect either HDR- or NHEJ-mediated integration of both Donor and CRISPR vectors. Integration of CRISPR vector genomes containing an intact *Cas9* transgene would be a particular concern, given the ongoing risk for off-target effects from ongoing CRISPR/Cas9 nuclease activity. This risk was somewhat ameliorated by the absence of detectable intact *Cas9* transgenes (Figure 6, F and G), and by the low frequency of detectable *Cas9* sequences in the liver (~0.3 copies/genome; Figure 4E). In contrast, multiple intact, integrated *G6PC* transgenes were detected, representing NHEJ-mediated integration of the therapeutic transgene. In contrast, HDR-mediated transgene integration could not be detected by the long-range sequencing method due to low depth of coverage for these events. Thus, no comparison for the frequency of NHEJ- versus HDR-mediated integration of the therapeutic transgene was possible.

Despite the absence of intact *Cas9* transgenes from long-range PacBio sequence data, we cannot rule out the NHEJ-mediated integration of large AAV concatamers that might contain the *Cas9* transgene. Long-range sequencing relies on PCR to amplify integrated vector genomes, and concatemerized AAV vector genomes would not be efficiently amplified. Future studies of genome editing for GSD Ia could avoid this risk by utilizing mRNA lipid nanoparticles to deliver *Cas9* (30).

This study was designed to identify conditions for genome editing that could be translated clinically to treat young patients with GSD Ia. The rapid loss of episomal AAV vector genomes from liver, especially in GSD Ia (3–6), preempts treatment of affected children early in life. Genome editing provides an alternative approach to genetic therapy, with high potential to treat stably, given that genetic modifications of hepatocytes will be impervious to cell division (17).

Three factors were optimized to increase suitability for clinical translation of the current genome editing approach: age of treatment, dose, and vector design. Infant mice were treated at 12 days of age, when the stage of development approximates that for human infants (hair present, eyes open) and clinical features of GSD Ia are present (3). The highest total vector dose was 1.9 × 10^13^ vg/kg, and even a 10-fold higher dose has been administered to human infants to treat spinal muscular atrophy through transduction of motor neurons (31). A higher clinical dose might be needed due to the lower tropism of AAV9 for human hepatocytes, in comparison with mice (32). In contrast, similar genome editing studies in neonatal mice at 2 to 3 days of age used vector doses of 1 × 10^14^ to 2 × 10^14^ vg/kg that could not be readily implemented in clinical trials (17–21). The vector design featured a functional *G6PC* transgene that is effective in GSD Ia due to both early expression from episomal Donor genomes and treating without regard to the pathogenic variants that cause GSD Ia.

The degree of correction in this study was sufficient to treat GSD Ia based on improved survival and improvement in blood glucose concentrations without complete normalization. Liver G6Pase reached 8% of normal, and HDR-mediated transgene integration was approximately 6% following administration of high-dose vectors with bezafibrate. The threshold for prevention of hypoglycemia and hepatocellular carcinoma in GSD Ia has been established at 3% of normal G6Pase (5, 26).

The observed HDR-mediated integration frequency of approximately 6% for 12-day-old infant mice with GSD Ia was higher than might have been expected given the age at treatment. The great majority of similar studies treated neonatal affected mice with various inherited metabolic disorders to accomplish genome editing of the liver (17–21), which have a higher rate of hepatocyte division that promotes HDR. A recent study of genome editing in neonatal mice with citrullinemia type I utilized a promoterless transgene and *Staphylococcus aureus* CRISPR/Cas9 to achieve up to 15% HDR-mediated transgene integration (33). Several studies documented lower-frequency genome editing when older mice were treated. For ornithine transcarbamylase deficiency, 10% gene correction was observed for neonatal mice, while adult treated mice had 0.3% correction (17). A study in mice with Wilson disease demonstrated 7% HDR-mediated integration when 3-week-old mice were treated in the presence of a selective advantage; however, WT mice treated in parallel had undetectable HDR (34). Mice with phenylketonuria had a baseline HDR-mediated integration frequency of 1%, which was increased to 13% by adding vanillin treatment to suppress the competing process of NHEJ. It is possible that GSD Ia mice feature a selective advantage for corrected cells, given the presence of a degree of hepatoxicity in absence of effective gene therapy as shown by increased liver transaminases (35). We observed such a selective advantage based on increased allele modification in GSD Ia mice, compared with their normal littermates, in a previous study of ZFN-mediated genome editing (12).

An alternative approach, termed GeneRide (23, 36), has been developed for genome editing in metabolic disorders, which delivers a donor vector containing a promoterless transgene (with or with CRISPR/Cas9) to integrate in the *Alb* locus and drive high hepatic expression. This strategy has successfully treated juvenile (37) and adult (38) mice with methylmalonic acidemia (MMA), which was possible despite a low rate of HDR in older mice due to a selective advantage for corrected hepatocytes in MMA (18, 37). While this strategy could be viewed as a strategy for treating inherited metabolic disorders, it would not be appropriate for GSD Ia due to the need for regulated expression that is provided by the endogenous promoter included in the transgenes expressing *G6PC* (39). Overexpression of *G6PC*, such as that from the *Alb* promoter, can produce a prediabetic state that should be avoided to prevent therapy-related toxicity (40).

Limitations of this study included a lack of age-matched controls due to high mortality for untreated *G6pc^–/–^* mice that require gene therapy to survive (41), and limited analysis of off-target activity of CRISPR/Cas9. A future long-term study could use a liver-specific *G6pc*-knockout mice that survives without administration of gene therapy, which would allow comparisons with untreated, affected control mice (42). Furthermore, off-target analysis for genome editing of the mouse genome has limited relevance to the eventual clinical translation of genome editing, when safety will be paramount and any off-target activity in the human genome must be understood. Future preclinical development of genome editing will include unbiased evaluation of off-target activity for relevant guide sequences in a humanized model of GSD Ia using established methods (43).

This study has shown that integration of a full-length transgene using CRISPR/Cas9-based genome editing is efficacious in GSD Ia, and that transgene integration is nuclease dependent. The strategy using 2 AAV vectors, including one that expresses G6Pase as an episome and integrates into the host genome, provides both immediate and sustained therapeutic benefits. Importantly, this approach will also treat all pathogenic variants in *G6PC*, regardless of the underlying pathogenic variant(s), and therefore designing multiple CRISPR constructs, and demonstrating their safety, is not required to treat the entire patient population. The demonstrated frequency of editing and transgene integration in the mouse model supports the continued development of genome editing for GSD Ia, especially in combination with drugs like bezafibrate that are known to increase gene expression from AAV vectors. Genome editing will likely be needed to stably treat patients with GSD Ia early in life. These data provide a model for the development of genome editing therapies for not only GSD Ia, but many other inherited metabolic disorders.

## Methods

### Sex as a biological variable.

GSD Ia is an autosomal recessive disorder that affects both sexes, and therefore both male and female mice were included in each group. Mice were treated as infants prior to the ability to assign sex, and the study was not adequately powered to detect differences in response based upon the biological variable of sex.

### AAV vector production.

The AAV9 serotype has been described previously (44). The AAV vector plasmid pAAV-CRISPR contained a vector gene containing a terminal repeat (TR) at each end flanking a minimal *G6PC* promoter expressing Cas9 from *S*. *pyogenes* with a FLAG tag and bovine growth hormone genomic polyadenylation sequence. The second AAV vector plasmid, pAAV-mDonor, contained a TR at each end flanking 2 transgenes: (a) the human *G6PC* cDNA flanked upstream by the homology arm the containing 5′ UTR genomic sequence of mouse *G6pc*, including a 297 bp minimal *G6PC* promoter, and downstream by the human growth hormone genomic polyadenylation sequence followed by the homology arm containing the intron 1 genomic sequence of mouse *G6pc*; and (b) the *U6* promoter expressing a gRNA specific for the exon 1/intron 1 boundary of *G6pc*. Vectors were packaged, purified, and quantified by Southern blotting as described previously (24).

### In vivo evaluation of genome editing.

Adult *G6pc*^+/–^ mice (gift from Janice Chou at the National Institutes of Child Health and Human Development) were bred to produce homozygous *G6pc^–/–^, G6pc*^+/–^, and *G6pc*^+/+^ offspring. *G6pc^–/–^* mice neonates are smaller with protuberant abdomens by 3 days of age, when daily subcutaneous injections of 0.1 to 0.2 mL 10% dextrose are initiated. Bezafibrate was administered starting at 5 days of age and continuing for 21 days. Bezafibrate was dissolved in 1:10 DMSO/PBS (1.2 mg/mL solution, dose 10 mL/g; 12 mg/kg/day dose). *G6pc^–/–^* mice were injected with vectors via the retro-orbital sinus at 12 ± 1 days of age. Injection was performed following isoflurane anesthesia with a 28-gauge insulin syringe, and hemostasis was achieved by brief manual pressure. An 8-hour fast was performed at 2 and 11 weeks following vector administration prior to obtaining a sample from a tail clip for blood glucose. Mice were euthanized for tissue collection 12 weeks following vector administration. Liver tissue was frozen on dry ice for biochemical analysis, and preserved in formalin for histology.

### Assessment of biochemical and molecular endpoints.

AAV vector genome copy number was measured by quantitative real-time PCR with liver genomic DNA and normalized to β-actin. Plasmid DNA corresponding to 0.01 to 100 copies of *G6PC* (in 500 ng genomic DNA) was used in a standard curve. qPCR was performed on a LightCycler 480 (Roche Diagnostics) using SYBR Green mix (Thermo Fisher Scientific) and the following primers: human *G6PC* Fwd (5′-GCAGTTCCCTGTAACCTGTGAG-3′), human *G6PC* Rev (5′-GGTCGGCTTTATCTTTCCCTG-3′), SpCas9 Fwd (5′-AGTACAGCATCGGCCTGGAC-3′), SpCas9 Rev (5′-GGGCTCCGATCAGGTTCTTC-3′), mouse β-actin Fwd (5′-GGCTGTATTCCCCTCCATCG-3′), and mouse β-actin Rev (5′-CCAGTTGGTAACAATGCCATGT-3′). Cycling conditions were 95°C for 5 minutes, followed by 45 cycles of 95°C for 10 seconds, 60°C for 10 seconds, and 72°C for 20 seconds followed by data acquisition.

Enzyme analysis was performed as described previously (3). Briefly, tissues were flash-frozen and stored at –70°C. Glycogen content was measured by complete digestion of polysaccharide using amyloglucosidase (Sigma Chemical Co.). The structure of the polysaccharide was inferred by using phosphorylase free of the debranching enzyme to measure the yield of glucose-1-phosphate. Specific G6Pase activity was measured by using glucose-6-phosphate as substrate after subtraction of nonspecific phosphatase activity as estimated by β-glycerophosphate.

Prevention of hypoglycemia was assessed by fasting the mice for 8 hours. Blood glucose was measured by a point-of-care glucometer, either the AlphaTRAK or AlphaTRAK2 (Zoetis).

The GTT was performed to assess glucose metabolism. All mice were given an intraperitoneal (i.p.) injection of 1 mg of dextrose per gram of body weight after a 4-hour fast. Tail clip blood samples were drawn during fasting prior to the i.p. injection, and again at 15, 30, 60, and 120 minutes after injection. This test provided information on the amount of glucose uptake into insulin-sensitive tissues throughout the course of the 120-minute postinjection period. Plasma glucose levels were analyzed by Alphatrak glucose monitoring (Zoetis).

### Evaluation of liver histology.

Vacuolation of hepatocytes was assessed by quantification from H&E-stained liver sections. All vacuolation counts were made using Fiji (ImageJ). Images from stained liver sections were first converted to 8-bit grayscale and then thresholded to 180:255. The built-in watershed function was applied. Finally, images were counted using the Analyze Particles within a range of 50–10,000 pixels. Fiji automatically displayed a total count of vacuoles along with the average size.

### Evaluation of nuclease activity and transgene integration.

The Surveyor Nuclease Detection Assay (Integrated DNA Technologies) was performed using purified DNA, the murine *G6pc* locus was PCR amplified using the primers mousesurveyorFwd (5′-TGACCTACAGACTGAATCCAGG-3′) and mousesurveyorRev (5′-TAACATCTGTGCTCAGGAGCTG-3′). The PCR product was analyzed using the Surveyor Mutation Detection Kit (Integrated DNA Technologies) according to the manufacturer’s instructions. The PCR products were also sequenced using Sanger sequencing methods (Eton Biosciences).

DNA integration quantification was performed as follows. A synthetic DNA fragment was generated by PCR with primers M1 (5′-CAGCCGCACAAGAAGTCGTTG-3′) and M4 (5′-TCTGGGAATCAGGGACTGGG-3′) in the first round of PCR, followed by primers M2 (5′-CCACTCCCACTGTCCTTTCC-3′) and M3 (5′-GGCTCAGTAGATCAAGTGCCTGC-3′). The PCR fragment contained the junction fragment from the 3′ end of the human *G6PC* cDNA in the transgene to the intron 1 *G6pc* sequence in mouse genomic DNA. Serial dilutions of the synthetic DNA templates were prepared and used as the starting template for each PCR reaction to generate the standard curve. Mouse genomic DNA was amplified simultaneously to measure the level of integrated transgene and the *G6pc* locus.

The web-based tool CRISPOR (https://crispor.gi.ucsc.edu/) was used to predict potential off-target sites for gRNAs. No off-target sites were found with no or 1 mismatch in the gRNA. With 2 mismatches, 9 target sites were identified, and all were in intronic (3) or intergenic (7) regions. With 3 mismatches, 2 exonic regions outside the target exon were identified. Primers were designed to target sites (Figure 5C). PCR products were extracted from the gel using the Qiagen Gel Extraction Kit (catalog 28704). Illumina NGS technology was used, and data were analyzed by the Azenta Lifesciences bioinformatics team. NGS sequencing of off-target sites 3, 5, 6, and 7 was complicated by the coamplification of paralogous genomic regions during PCR. To remove paralog amplicons, fastq files for sites 3, 5, 6, and 7 were first aligned against the GRCm38 reference and then to the target amplicon using CRISPRessoPooled analysis (https://docs.crispresso.com/suite/pooled/tool.html).

### Assessment of immune responses.

ELISA determination of a murine anti-SpCas9 humoral response was performed with recombinant SpCas9 (catalog CAS9PROT, MilliporeSigma), which was diluted to 0.05 μg/mL in 1× PBS and used to coat a 96-well MaxiSorp plate (catalog 442404, Thermo Fisher Scientific). Wells were coated overnight at 4°C with 100 μL/well of coating solution. All subsequent incubations were carried out at room temperature on an orbital shaker and all wash steps were repeated 3 times. Plates were then washed with 1× PBS with 0.05% Tween 20 (PBST). Plates were blocked for 2 hours with 200 μL/well of PBST plus 3% w/v nonfat dry milk (catalog 1706404, Bio-Rad). Dilutions of heat-inactivated mouse serum were prepared in PBST with 1% w/v BSA (catalog AP-4510-80, SeraCare) in a separate plate. Serum diluted 1:100 was then added to the plate at 100 μL/well and incubated for 1 hour. Plates were then washed with PBST. Goat anti-mouse IgG HRP-conjugated secondary antibody (catalog 1030-05, SouthernBiotech) was diluted 1:1000 in PBST. Secondary antibody solution (100 μL) was added per well and incubated for 30 minutes. Plates were washed a final time with PBST. Plates were developed for 30 minutes with 50 μL/well of TMB ELISA substrate (catalog 34028, Thermo Fisher Scientific). Development was stopped by the addition of 50 μL/well of 0.16 M sulfuric acid (catalog N600, Thermo Fisher Scientific). Absorbance was recorded at 450 nm for all samples.

The positive control for the SpCas9 ELISA was generated by vaccination with recombinant SpCas9 (catalog CAS9PROT, MilliporeSigma), which was purchased as lyophilized protein and reconstituted in sterile 0.9% saline (catalog NDC 0409-4888-03, Hospira) to a concentration of 1 μg/μL. Thirty microliters of SpCas9 solution was mixed 1:1 with room temperature Addavax adjuvant (catalog vac-adx-10, Invivogen) via pipet. Twenty-five microliters of SpCas9/Addavax solution was injected into each gastrocnemius of a 10-week-old, male *G6pc*^+/–^ mouse for a total dose of 50 μL. Blood was collected via cheek bleed immediately before vaccination and 2 weeks after vaccination. Serum was isolated using a serum separator tube (catalog 450472, Thermo Fisher Scientific) via standard protocol. Collected serum was heat inactivated at 56°C for 30 minutes and then stored frozen until analysis.

CD3^+^ lymphocyte staining was performed with a rabbit monoclonal antibody against CD3 (catalog RM-9107s, Thermo Fisher Scientific). Anti-CD3 was used at 1:50, with Discovery Ab Diluent (catalog 760-108, Roche). Immunohistochemical tests were performed using the Ultra Discovery automated staining platform. The tissue sections were pretreated (epitope retrieval) with cell conditioning solution CC1 (catalog 950-124, Roche) for 56 minutes. Anti-CD3 was applied and incubated for 60 minutes at 37°C. Rabbit IgG, substituted for the primary antibody, was used as the negative control. After binding of the primary antibody, anti–rabbit HQ (catalog 760-4815, Roche) was applied and incubated for 12 minutes, followed by 12 minutes of incubation with HRP-conjugated secondary antibody. For visualization, Discovery ChromoMap DAB (catalog 760-159, Roche) was applied and incubated for 5 minutes. Immunoreactive lymphoid cells were counted in 10 random fields at ×400 magnification by a pathologist using an Olympus BX51 microscope.

### Long-read PacBio sequencing of transgene integration events.

To further characterize the editing outcomes in GSD Ia mice, we analyzed amplicons spanning the mouse *G6pc* gRNA target site using the approach recently described by Giorgi et al. (45). For this method, we isolated genomic DNA from mouse livers and performed 2 cycles of PCR amplification with primers spaced approximately 750 bp from the gRNA target site and which contained an 18-bp UMI flanked by a 24-bp synthetic priming site (PCR1). Forward primer: CAAGCAGAAGACGGCATACGAGATNNNYRNNNYRNNNYRNNNCACTGTTGGCTGCCCAAAC; Reverse Primer: AATGATACGGCGACCACCGAGATCNNNYRNNNYRNNNYRNNNGCCTGGCACATAGTAGCTGA. Dual-UMI-tagged amplicons were then amplified by an additional 25 rounds of PCR using the synthetic priming sites (PCR2), followed by an additional 10 rounds of PCR using the synthetic priming sites with barcoded primers (PCR3) to facilitate multiplexing samples. All PCR reactions were performed with LongAmp Hot Start Taq DNA Polymerase (New England Biolabs, catalog M0534L) according to the manufacturer’s recommendations, with a 65°C annealing temperature and 18-minute extension time. After PCR1, excess UMI containing oligos were degraded by treatment with Exonuclease I (New England Biolabs, catalog M0568L). After exonuclease treatment, and again after PCR2 and PCR3, amplicons were purified by SPRIselect beads (Beckman Coulter, catalog B23317) using a 0.5× bead ratio. After the final purification, the HiFi library was prepared from 1 μg of pooled, barcoded amplicons and sequenced on a PacBio Revio instrument.

Demultiplexing of CCS reads was performed with cutadapt v5.0 (http://dx.doi.org/10.14806/ej.17.1.200). Generation of consensus UMI sequences, adapter trimming, and PCR chimera screening were performed with the long-read UMI pipeline (PMID: 33432244). Nontarget reads were removed by excluding those lacking alignment to the first and last 100 bp of the expected *G6pc* amplicon. Classification of editing events was performed with CRISPResso v2.3.2 using a minimum identity score of 10% to account for large insertions (PMID: 30809026). The classification of large insertions was performed by manual review.

### Statistics.

Statistical analysis was performed using GraphPad Prism 10. Statistical significance was determined by 1-way ANOVA with Dunnett’s multiple-comparison test, or by 2-tailed homoscedastic *t* test for comparisons of 2 groups. A *P* value of less than 0.05 was considered significant.

### Study approval.

This study was approved by the Duke Institutional Animal Care and Use Committee (Duke University School of Medicine).

### Data availability.

Detailed information for each base-editing vector is available from GenBank. The accession numbers are as follows: CRISPR vector plasmid, PV925756; Donor vector plasmid, PV925757. PacBio sequencing data are accessible at the NCBI Sequence Read Archive (SRA) under accession PRJNA1292206. Values for all data points in graphs are reported in the Supporting Data Values file.

## Author contributions

BA and DDK performed experiments, analyzed data, and wrote the manuscript. EI performed experiments, analyzed data, and edited the manuscript. TVB developed and performed the ELISA for Cas9, and the long-range PacBio sequencing. SL, DG, MW, SYC, and HRK performed experiments and analyzed data. EDB provided veterinary care, performed experiments, analyzed data, and edited the manuscript. GH analyzed hepatocyte vacuolation. DC provided technical advice and edited the manuscript. JIE provided analysis of histology. BRC provided technical advice, shared plasmids for construction of the CRISPR/Cas9 vector, and edited the manuscript.

## Supplementary Material

Supplemental data

Unedited blot and gel images

Supporting data values

## Version 1 07/31/2025

In-Press Preview

## Version 2 09/23/2025

Electronic publication
