# Supplementary material for: Efficacious genome editing in infant mice with glycogen storage disease type Ia
Source: JCI Insight. 2025 Jul 31;10(18):e181760. doi: 10.1172/jci.insight.181760 (PMC12487852; doi:10.1172/jci.insight.181760)
Supplement: Supplemental data [file jciinsight-10-181760-s259.pdf]

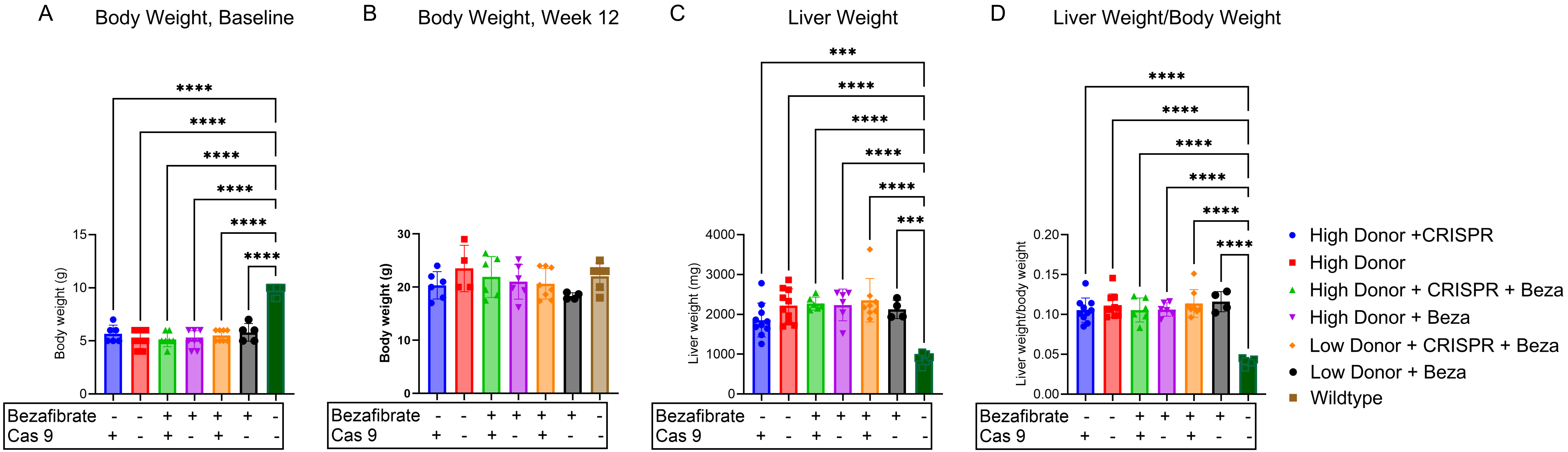

Supplemental Figure 1: Weight following treatment. (A) Body weight 2 weeks and (B) 12 weeks after vector administration. (C) Liver weight, and (D) liver weight/body weight. Individual values and mean are shown. P values indicated as follows: ** = p<0.01; **** = p<0.0001, compared with High D+C Beza group as determined by one-way ANOVA with Dunnett's multiple comparisons test.

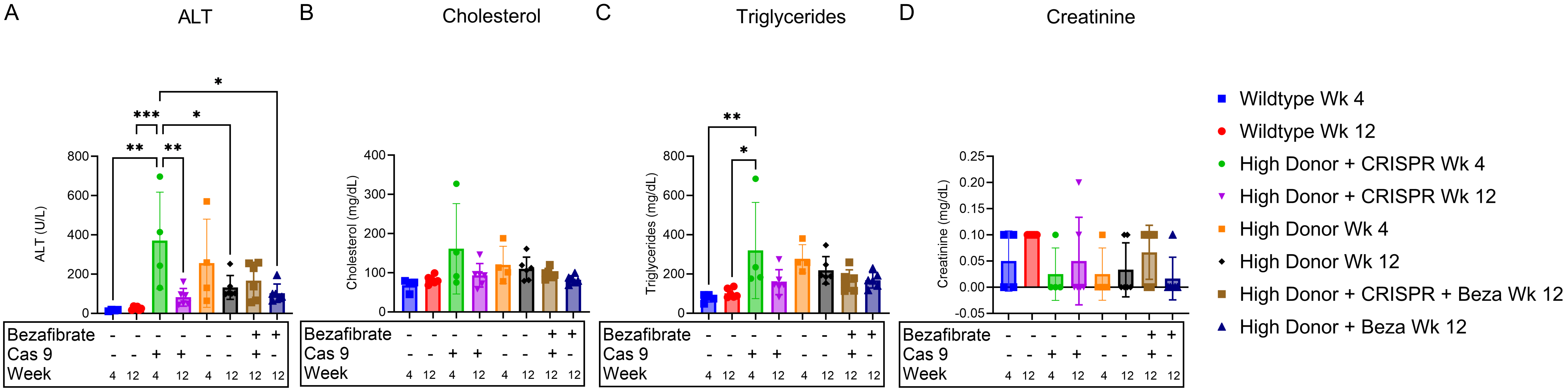

Supplemental Figure 2: Biomarker testing 4 and 12 weeks following treatment. (A) ALT, (B) cholesterol, (C) triglycerides, and (D) creatinine. Individual values and mean are shown. P values indicated as follows: ** = p<0.01; **** = p<0.0001, compared with High D+C Beza group as determined by one-way ANOVA with Dunnett's multiple comparisons test.

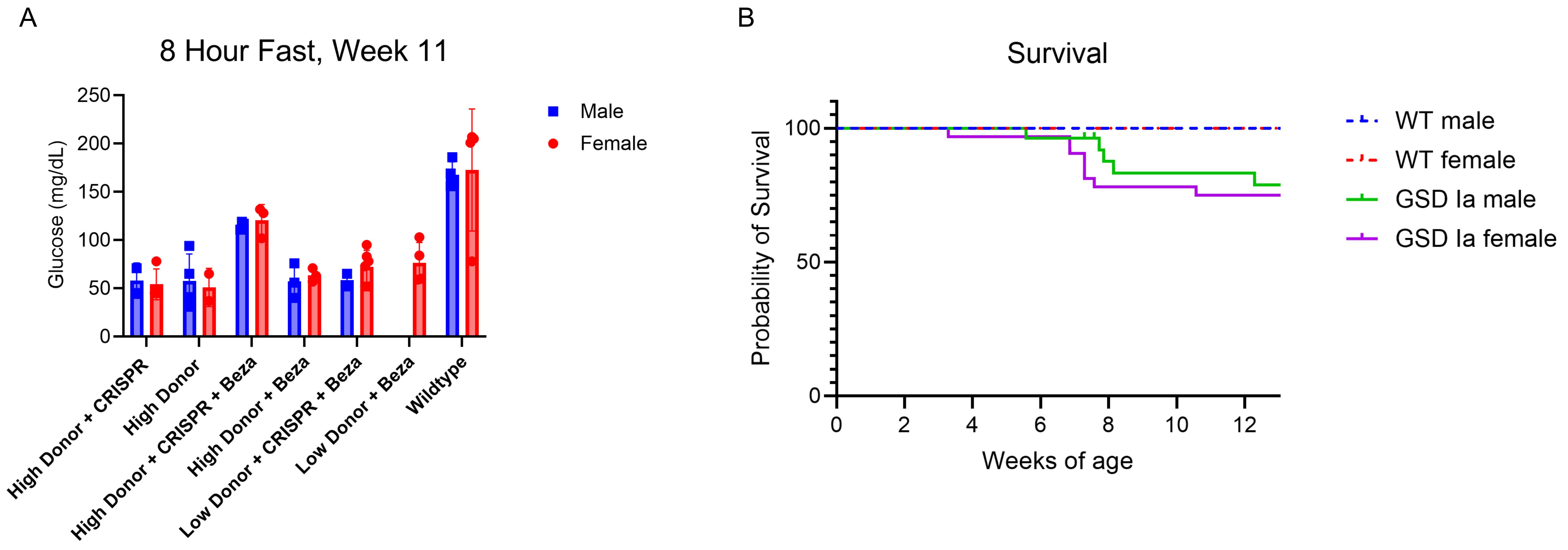

Supplemental Figure 3: Sex as a biological variable. (A) Blood glucose following an 8 hour fast, 11 weeks following vector administration. Individual values and mean are shown. (B) Survival. Kaplan-Meier survival analysis.

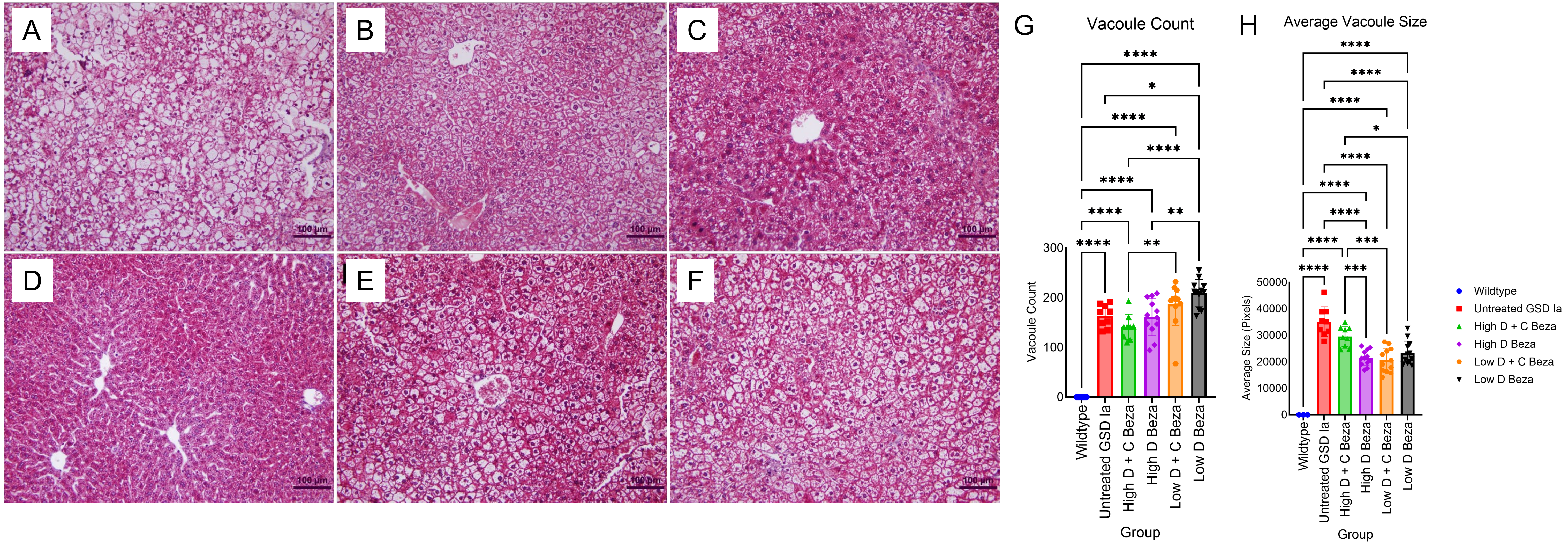

Supplemental Figure 4. Liver vacuolation following genome editing. Photomicrographs of mouse liver 200x, Masson's Trichrome Stain. A: Untreated GSD Ia, B: High D+C Beza, C: High D Beza, D: WT, E: Low D+C Beza, F: Low D Beza. Vacuolation was quantified in liver sections. The number of vacuoles (G) and vacuole size (in pixels) (H) of liver histology samples from wild type mice (n=3) and GSD Ia treatment groups: GSD Ia Untreated (UT; n=3), High D+C Beza (n=3), High D Beza (n=4), Low D+C Beza (n=4) and Low D Beza (n=4). All images were all taken at equal resolution and magnification. Three representative regions of interest from H&E samples were quantified per mouse. Data is represented as Mean +-SD. Significance was validated with one-way ANOVA test.

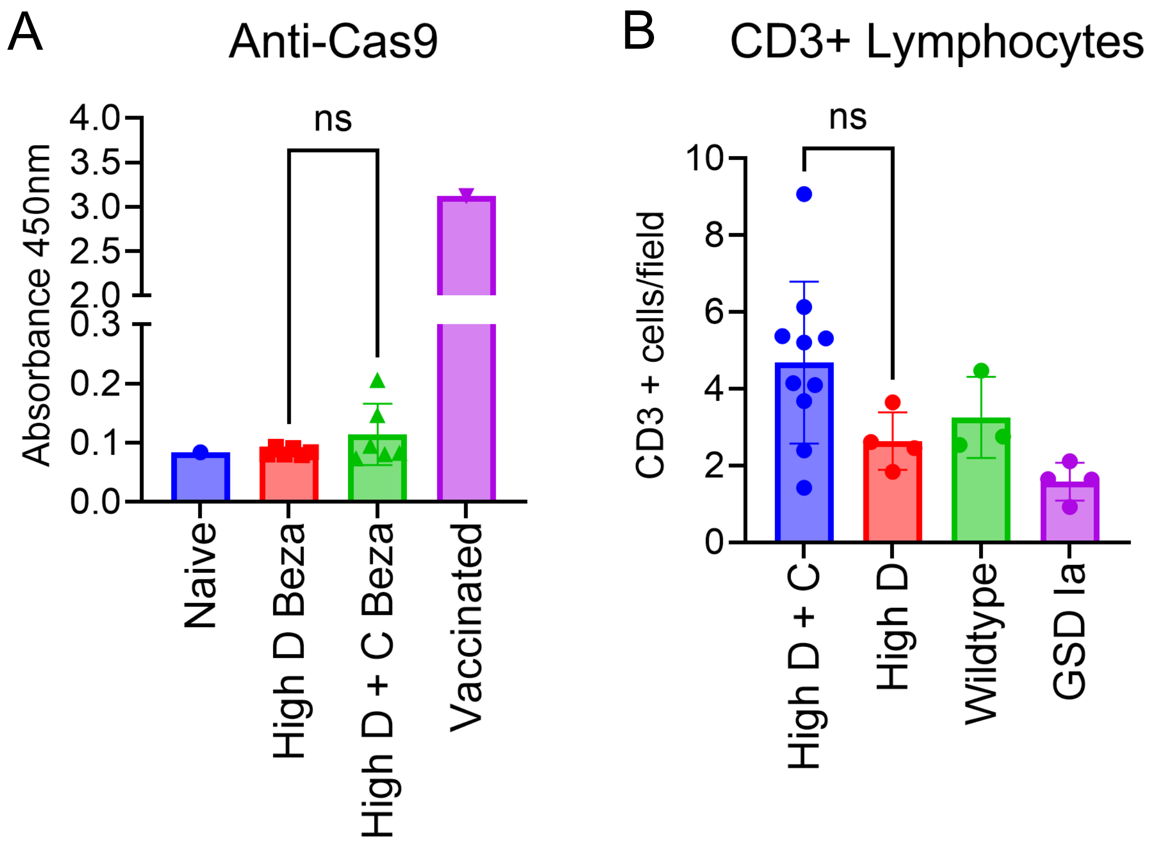

Supplemental Figure 5: **ELISA for anti-SpCas9 IgG in GSD Ia mice after vector administration.** (A) Serum from mice either treated with High dose Donor and CRISPR vectors and bezafibrate (High D+C Beza; n = 6) or Donor vector with bezafibrate (H D Beza; n = 6) was collected 12 weeks post-treatment and analyzed by indirect ELISA for SpCas9 reactive antibodies. 450nm absorbance values are plotted for serum tested at a 1:100 dilution. Dots represent values from individual mice. As assay controls, serum from a GSD Ia carrier mouse pre- and post-vaccination with SpCas9 was included (n=1). Statistical analysis was performed using Welch's *t*-test to compare only the absorbance of Donor + CRISPR and Donor only groups. *P* value ns = not significant. (B) CD3 staining of liver sections was performed 4 weeks post-treatment from mice treated with High dose Donor and CRISPR vectors (High D+C; n=10) or High dose Donor vector (High D; n=4). Wildtype age-matched and 12 day old, untreated GSD Ia liver samples were included as controls. Statistical analysis with One-way ANOVA. *P* value ns = not significant.
