# Supplementary figures and images for: Efficacious genome editing in infant mice with glycogen storage disease type Ia
Source: JCI Insight. 2025 Jul 31;10(18):e181760. doi: 10.1172/jci.insight.181760 (PMC12487852; doi:10.1172/jci.insight.181760)

Figure 5A. Intact Gel

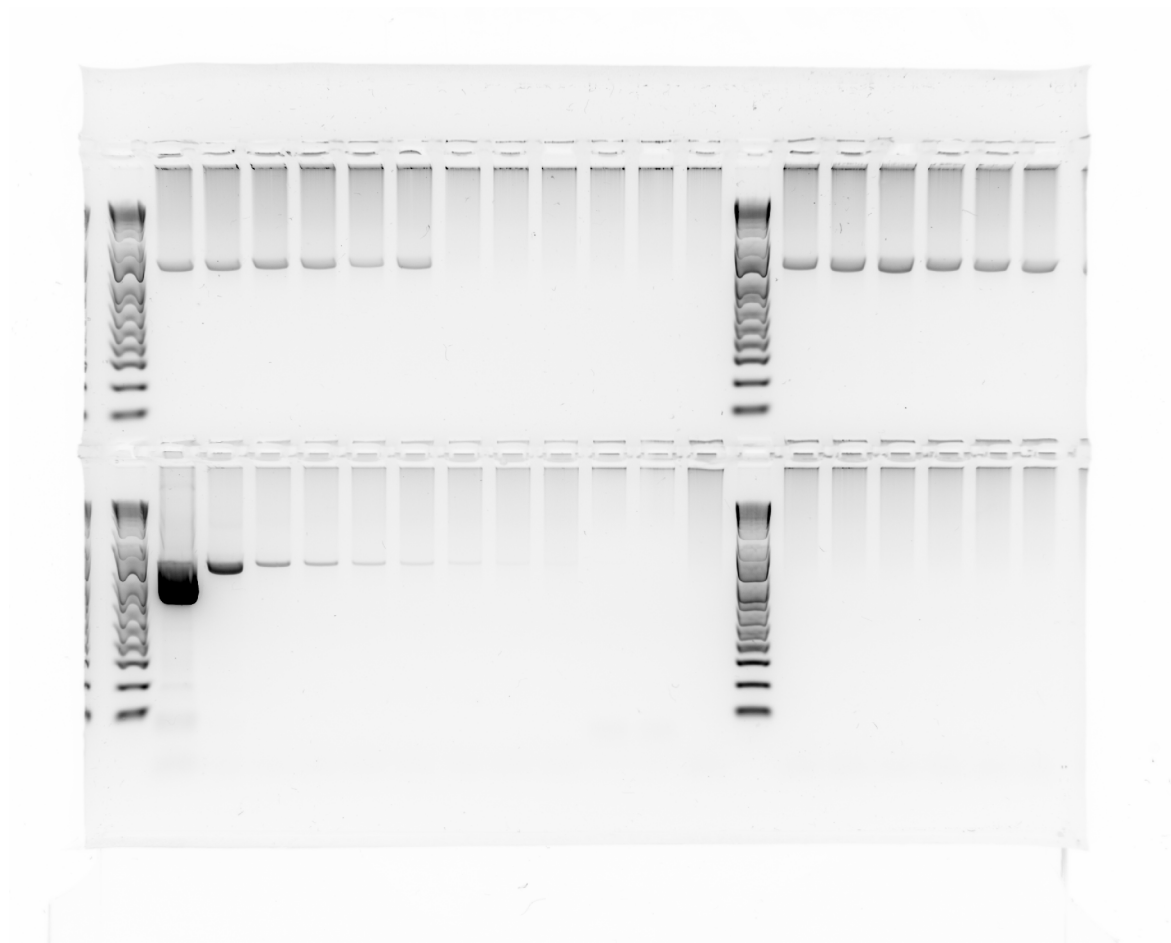

Figure 5B. Intact Gels

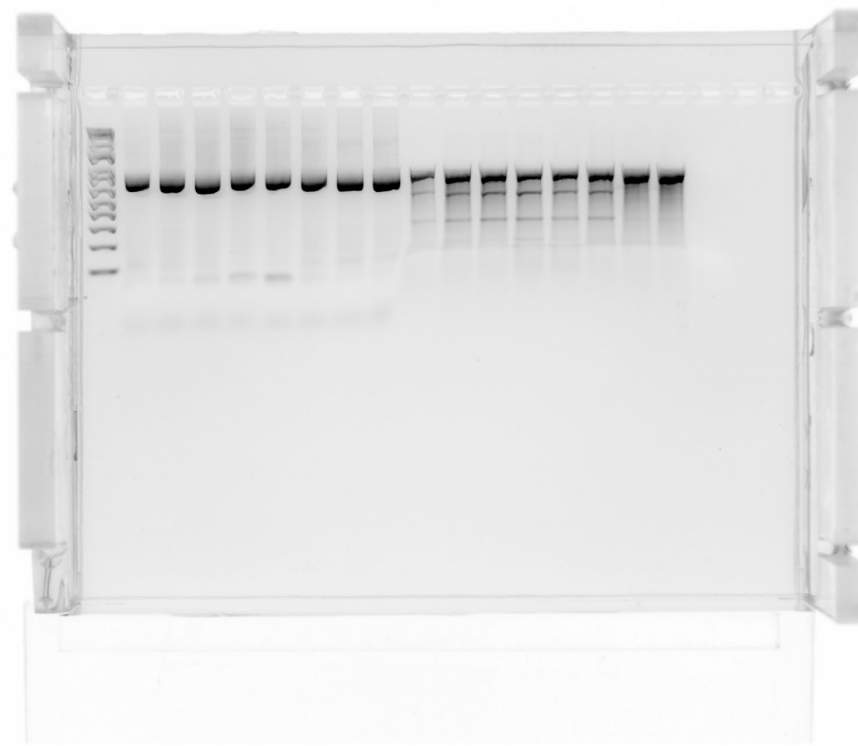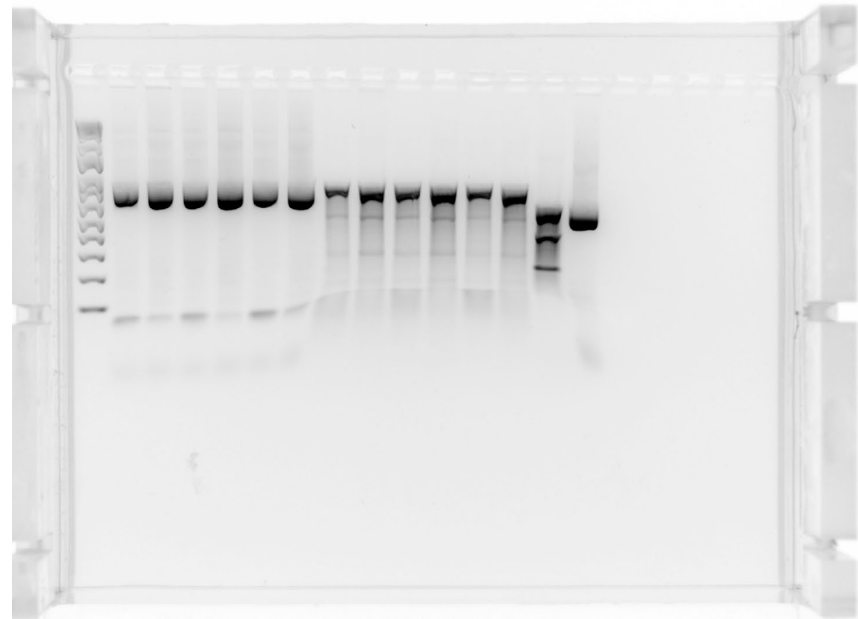

Supplement: Unedited blot and gel images [file jciinsight-10-181760-s260.pdf]
